# Motivations for contralateral prophylactic mastectomy as a function of socioeconomic status

**DOI:** 10.1186/s12905-017-0366-2

**Published:** 2017-02-01

**Authors:** Dadrie F. Baptiste, Erina L. MacGeorge, Maria K. Venetis, Ashton Mouton, L. Brooke Friley, Rebekah Pastor, Kristen Hatten, Janaka Lagoo, Susan E. Clare, Monet W. Bowling

**Affiliations:** 1 Department of Surgery, Indiana University School of Medicine, 545 Barnhill Drive, Emerson Hall 202, Indianapolis, IN 46202 USA; 2 Department of Communication Arts and Sciences, Pennsylvania State University, 234 Sparks Building, University Park, PA 16802 USA; 3 Brian Lamb School of Communication, Purdue University, BRNG 2264, 100 North University Street, West Lafayette, IN 47907-2098 USA; 4 Department of Surgery, Feinberg School of Medicine, Northwestern University, 303 E. Superior Street, Lurie 4-113, Chicago, IL 60611 USA; 5 Present address: William Beaumont Hospital, 3601 W 13 Mile Rd, Royal Oak, MI 48073 USA; 6 Present address: Department of Communication and Media, Texas A&M—Corpus Christi, 6300 Ocean Dr., Corpus Christi, TX 78412 USA; 7 Present address: CoreClarity, PO Box 863692, Plano, TX 75086 USA; 8 Present address: School of Communication, Western Michigan University, 1903 W Michigan Ave., Kalamazoo, MI 49008 USA; 9 Present address: Ariadne Labs, 401 Park Drive, Boston, MA 02215 USA; 10 Present address: Hendricks Regional Health, 1000 East Main Street, Danville, IN 46122 USA

**Keywords:** Breast cancer, Contralateral prophylactic mastectomy, Motivation, Socioeconomic

## Abstract

**Background:**

Despite no demonstrated survival advantage for women at average risk of breast cancer, rates of contralateral prophylactic mastectomy (CPM) continue to increase. Research reveals women with higher socioeconomic status (SES) are more likely to select CPM. This study examines how indicators of SES, age, and disease severity affect CPM motivations.

**Methods:**

Patients (*N* = 113) who underwent CPM at four Indiana University affiliated hospitals completed telephone interviews in 2013. Participants answered questions about 11 CPM motivations and provided demographic information. Responses to motivation items were factor analyzed, resulting in 4 motivational factors: reducing long-term risk, symmetry, avoiding future medical visits, and avoiding treatments.

**Results:**

Across demographic differences, reducing long-term risk was the strongest CPM motivation. Lower income predicted stronger motivation to reduce long-term risk and avoid treatment. Older participants were more motivated to avoid treatment; younger and more-educated patients were more concerned about symmetry. Greater severity of diagnosis predicted avoiding treatments.

**Conclusions:**

Reducing long-term risk is the primary motivation across groups, but there are also notable differences as a function of age, education, income, and disease severity. To stop the trend of increasing CPM, physicians must tailor patient counseling to address motivations that are consistent across patient populations and those that vary between populations.

**Electronic supplementary material:**

The online version of this article (doi:10.1186/s12905-017-0366-2) contains supplementary material, which is available to authorized users.

## Background

Despite the lack of a demonstrated survival advantage, the rate of contralateral prophylactic mastectomy (CPM) for the treatment of unilateral breast cancer in women without a genetic predisposition for the development of breast cancer continues to increase unabatedly [1–7]. Increasing use of this procedure contradicts expert medical recommendations, such as those of The National Comprehensive Cancer Network; indeed, it has been estimated that fewer than 10% of women with newly diagnosed breast cancer meet the clinical criteria for CPM [8]. Reversing this trend requires that we understand why women are choosing CPM.

A 2011 survey of CPM-related research noted that women’s reasons for electing CPM were poorly understood [9]. Research conducted in the interim indicates that women choosing CPM are not being influenced by the medical evidence demonstrating no survival advantage [8, 10, 11]. Instead, substantial numbers of women believe they are at considerable risk of a new contralateral primary, and CPM improves their likelihood of survival [10, 11]. Even in the extreme case of the diagnosis of ductal carcinoma in situ (DCIS, Stage 0) this holds true. Breast cancer specific survival for women diagnosed with DCIS and treated by breast conservation or unilateral mastectomy is on the order of 99% at 20 years [12]. Nevertheless, SEER data documents an increase in CPM for DCIS patients from 12.7% in 1998 to 36.5% of cases in 2011 [13]. Patients also report seeking CPM to avoid subsequent medical visits and treatments (e.g., surgery, chemotherapy, medications), and to retain/obtain a symmetrical appearance [10, 11, 14–16].

At the same time, it is clear that the choice of CPM is not uniform across groups of women in the United States. Data from the National Cancer Data Base [17] and the Florida Cancer Data System [18], show that higher socioeconomic status (SES) and private insurance are positively associated with undergoing CPM. Correspondingly, White women undergo CPM at a rate at least twice that of Black women [8]. These factors may be proxies for access to all forms of medical care, but they also may represent variation in sources of information about cancer, differing levels of concern about body image, or differing risk perceptions [19, 20]. For example, multiple studies have shown that White women are more likely than other racial groups to overestimate their risk of developing breast cancer [21, 22]. Another demographic associated with higher CPM rates is young age at diagnosis [23–25]. The decision of younger patients to avail themselves of CPM has been attributed to the expectation of a long life following the initial diagnosis with a consequently higher lifetime risk of developing another primary breast cancer [15, 23]. Most prior studies of reasons for CPM have focused on small subsets of possible motivations [8, 26], often reporting percentages of patients experiencing particular motivations or concerns [11]. Alternatively, qualitative studies have explored patients verbatim explanations about the factors that influenced them [10]. Such studies have not directly addressed variation in motivations among patients with different socioeconomic and demographic characteristics. To remedy limitations in the existing research and provide additional insights into CPM decision making, this study addresses the following research questions: (1) What personal motivations for seeking CPM are stronger or weaker overall, and (2) to what extent does their importance for women vary as a function of education, employment, income, insurance status, race, age, and disease severity?

## Methods

### Ethics

This study was approved by the Indiana University Institutional Review Board (IRB-04, protocol number 1210009689; and IRB-03, protocol number 1304011094). All research was carried out in compliance with the Helsinki Declaration.

### Questionnaire development

The literature was consulted in order to create questions that would adequately represent the range of patient motivations for CPM [10, 11, 14–16]. Draft versions of the questionnaire were piloted with four focus groups of five individuals each; one group consisted of lay individuals, one of physicians practicing non-surgical specialties, and two of the focus groups were made up of breast patients with five breast cancer survivors divided between these two groups. Based on their input, the questionnaire was modified by replacing scientific/medical terms with their lay equivalents, and removing what participants considered to be leading questions and questions that elicited a significant emotional response. The questionnaire is provided in Additional file 1.

### Data collection

This study was facilitated by access to patients who underwent CPM at four hospitals in Indianapolis, all of which are teaching institutions within the Indiana University School of Medicine. Wishard Memorial Hospital (now known as The Sidney and Lois Eskenazi Hospital) is a large public safety net hospital, with a patient population approximately equally divided between Black and non-Hispanic White individuals; most are underinsured. IU Health University Hospital is a tertiary referral center at which many patients have private health insurance. IU Health North and IU Health West are community hospitals. The same surgeons staffed the breast surgical oncology clinics at all four hospitals during the period of this study, helping to ensure that surgeon effects on CPM motivations and decisions were relatively constant across the sample.

Potential participants for this study were identified from hospital billing records using the procedure code for bilateral mastectomy during the years 2008–2012. The lists were then curated to identify patients who had undergone CPM (*n* = 326). Patients’ names, addresses, telephone numbers and the hospitals at which CPM was performed were obtained from the medical health record system. Approximately one-half of the patients were treated at IU Health University Hospital, slightly over a quarter at IU Health North and the remaining patients divided between IU Health West and Wishard. During 2013, these patients received a mailed introductory letter containing study information. Research team members included faculty, residents, and graduate students, all trained in research ethics and study procedures. Team members telephoned participants, obtained their verbal informed consent, and conducted structured interviews using the motivations questionnaire (*n* = 117) along with obtaining qualitative data that included brief narratives of their breast cancer experience (reported in [27]). Only 16 patients contacted were recorded as explicitly refusing the interview and information about these patients was not retained. These 16 patients declined participation for a variety of reasons. Some were still undergoing treatment and reported they were too emotionally “raw” to discuss their CPM decisions, some had experienced subsequent disease progression, and others wished not to revisit their illness. Other nonparticipation resulted from inability to reach patients at the phone numbers in their records.

### Study population

Patients who elected CPM to treat unilateral breast cancer completed a structured telephone interview. Participant demographics reflect participant status at the time of the surgery, and details are reported in Table 1. All participants were women, with an average age at diagnosis of 50, predominantly married and employed. A majority had private insurance; education and income varied. Most participants (*n* = 99, 88%) identified as White, with the remainder Black (*n* = 12, 11%) and Hispanic (*n* = 2, 1.8%). The average time between CPM surgery and survey completion ranged from 1 to 5 years, M = 3.07, SD = 2.26. Patient-reported diagnoses included invasive ductal cancer (*n* = 31, 27.4%), invasive cancer (*n* = 26, 23%), ductal carcinoma in situ (*n* = 23, 20.4%), mass/lump (*n* = 11, 8%), and other (*n* = 13, 11.5%). When describing their breast cancer experiences, four participants initially diagnosed prior to 2008 reported having had a CPM following a local recurrence. Four other patients self-identified as BRCA positive, and their data was removed from analysis because this suggests that CPM was medically indicated, changing the nature of the decision-making process.

**Table 1:** Participant characteristics

Diagnosis	
Invasive ductal cancer (*n* = 31)	27.4%
Invasive cancer (*n* = 26)	23.0%
Ductal carcinoma in situ (*n* = 23)	20.4%
Mass/lump (*n* = 11)	9.7%
Invasive lobular cancer (*n* = 9)	8.0%
Other (*n* = 13)	11.5%
Diagnosis dichotomy (*n* = 113)	
Less serious (*n* = 47)	41.6%
More serious (*n* = 66)	58.4%
Income	
Less than 25 K (*n* = 7)	6.2%
25–50 K (*n* = 20)	17.7%
51–75 K (*n* = 25)	22.1%
76–100 K (*n* = 17)	15.0%
101 K (*n* = 30)	10.6%
Declined to Answer (*n* = 14)	12.3%
Income dichotomy (*n* = 99)	
Less than 51 K (*n* = 27)	23.9%
51 K + (*n* = 72)	63.7%
Insurance	
Private (*n* = 80)	71.4%
Medicare/Medicaid (*n* = 12)	10.8%
Other (*n* = 13)	11.5%
Declined to Answer (*n* = 7)	6.3%
Insurance dichotomy (*n* = 105)	
Private (*n* = 80)	76.1%
Other (*n* = 25)	23.8%
Race/ethnicity	
White (*n* = 99)	87.6%
Black (*n* = 12)	10.6%
Hispanic (*n* = 2)	1.8%
Race/ethnicity dichotomy	
White (*n* = 99)	87.5%
Minority (*n* = 14)	12.5%
Marital status	
Married/partnership (*n* = 86)	76.8%
Divorced/separated (*n* =12)	10.7%
Single (*n* = 8)	7.1%
Widowed (*n* = 6)	5.4%
Education	
High school or less (*n* = 22)	19.5%
Some college (*n* = 36)	32.1%
Bachelor’s degree (*n* = 32)	28.6%
Graduate degree (*n* = 21)	18.8%
Declined to Answer (*n* = 2)	1.8%
Education dichotomy (*n =* 110)	
Less than BA (*n* = 58)	51.3%
BA+ (*n* = 53)	46.9%
Employment status	
Employed (*n* = 73)	64.6%
Retired (*n* = 18)	15.9%
Homemaker (*n* = 14)	12.4%
Unemployed (*n* = 4)	3.5%
Other (*n* = 1)	0.9%
Declined to Answer (*n* = 3)	1.8%
Employment dichotomy (*n =* 111)	
Employed (*n* = 73)	64.6%
Unemployed (*n* = 37)	32.7%
Age at Diagnosis	
Range 22-73, M = 50.29	
SD = 12.50	
Age dichotomy (*n* = 112)	
Younger than 46 (*n* = 44)	38.9%
46 and older (*n =* 68)	61.1%

Note. *N* = 113, except as indicated

Given the sample size, the variables of interest were dichotomized for analysis. Sample sizes for dichotomized groups are reported in Table 1. Using the dichotomization of Grimmer et al. [17], patients aged 45 or younger were classified as younger, with patients 46 and above classified as older. Patients who reported their diagnoses as invasive (ductal, lobular, or other) were classified as having more severe diagnoses, whereas patients with other diagnoses (e.g., DCIS, mass/lump, mammogram abnormality) were classified as having less severe diagnoses. Patients were also grouped into less educated (had not attained a bachelor’s degree) and more educated (bachelor’s or higher), and paid or unpaid work. A lower income group was created from patients who indicated that their income was in the categories 0-$25 K or 25 K–50 K; patients in any of the three higher-income categories were similarly grouped together. Underinsured patients were defined as all patients without commercial insurance or HMO coverage. At the Wishard and University Hospitals, such patients were covered by Medicare, Medicaid, or the Wishard Advantage assistance program. Wishard Advantage is funded by taxes from Marion County, which includes metropolitan Indianapolis, and helps provide coverage for patients with limited financial resources who do not qualify for Medicare or Medicaid. Race was classified as minority (Black and Hispanic) or majority (White, non-Hispanic). Because the number of minority participants was small, resulting in problematic statistical power and interpretation, we elected not to pursue our research question with regard to race, and instead treated race as a control variable in the subsequent analyses.

### Study measures

Participants were asked to consider their experience of 11 motivations for undergoing CPM. These were (1) *risk reduction—*belief that removing both breasts reduces likelihood of cancer coming back, (2) *recurrence-opposite*—concern that cancer would appear in the opposite breast, (3) *survival*—belief that removing both breasts increases the chance of long-term survival, (4) *surgery*—reduce or avoid need for later breast surgeries, (5) *symmetry*—desire to maintain a symmetrical appearance, (6) *recurrence-distant*—concern that cancer would appear elsewhere in the body, (7) *radiation*—desire to reduce or avoid need for radiation treatment, (8) *procedures*—desire to reduce or avoid procedures such as mammograms and MRIs, (9) *chemotherapy*—desire to reduce or avoid chemotherapy, (10) *physician visits*--desire to reduce or avoid future visits to the breast surgeon, oncologist, or hospital, and (11) *medications*-- desire to reduce or avoid medications that block hormones. Each motivation was assessed with a single-item measure, on a scale from *not at all* (1) to *a very large amount* (5). Age at diagnosis and diagnosis were assessed prior to the motivations; race, education, employment, income, and insurance were assessed afterward.

To determine whether the 11 motivation items represented a smaller set of dimensions, the 11 items were subjected to a principal components exploratory factor analyses with varimax rotation, computed in SPSS 23. Four factors were detected (all factor loadings > .76): avoiding treatment, reducing long-term risk, avoiding future visits, and symmetry. The avoiding treatment factor was comprised of items on the desire to reduce or avoid chemotherapy, radiation, and hormone blocking medication (λ = 2.22; α = .83, M = 2.14, SD = 1.28). The reducing long-term risk factor was comprised of items on the desire to avoid opposite breast new primary, risk reduction, and survival (λ = 2.3; α = .85, M = 4.2, SD = 0.91). The avoiding future visits factor was comprised of items on the desire to avoid physician visits and procedures (λ = 1.69; α = .81, M = 2.24, SD = 1.24). The single symmetry item loaded on its own factor (M = 3.42, SD = 1.44). Two items, assessing the recurrence-distant and surgery motivations, did not load onto any factors and consequently were not analyzed further.

### Statistical analyses

Data was analyzed in three stages. The first stage examined the relative strength of CPM motivations for all participants employing paired-sample t-tests. The second stage examined relationships between dichotomized predictor variables and CPM motivations, also using paired sample t-tests. Finally, the third stage utilized hierarchical multiple regression analyses to determine which predictors had effects independent of the others. For all analyses, unless otherwise specified, significance is reported at *p* < .05.

## Results

The first stage of the analysis examined the average endorsement of each motivation in the total sample. There was variation in the extent to which respondents endorsed the 4 motivation factors for CPM, ranging from strongest endorsement for reducing future risk (*M* = 4.2; 4 = *a large amount*) to weakest endorsement for avoiding future visits (2.2; 2 = *a small amount*). A series of paired-sample *t* tests revealed that the strength of reducing future risk was significantly greater than the other three motivations, and strength of symmetry was significantly stronger than both avoiding treatment and avoiding future visits. The strength of endorsement for avoiding treatment and avoiding future visits was not significantly different.

The second univariate stage of analysis compared the endorsement of the four motivation factors for (1) less severe versus more severe diagnosis, (2) younger versus older, (3) less and more educated, (4) paid versus unpaid employment, (5) lower and higher income, and (6) private versus other insurance. Mean comparisons are presented in Table 2. Respondents with a more severe diagnosis endorsed avoiding treatment more strongly than those with a less severe diagnosis (*p* < .01), and those with a less severe diagnosis more strongly endorsed reducing future risk (*p* = .06). Older respondents endorsed avoiding treatment more strongly than younger respondents (*p* < .01); younger respondents endorsed symmetry more strongly than older respondents (*p* < .05). Compared to less educated women, more educated women endorsed symmetry (*p* < .01). Compared to women with paid employment, women without paid employment endorsed avoiding treatment (*p* = .06). Women with less income endorsed reducing future risk more strongly than did women with more income (*p* < .01). Women with private insurance did not differ from women with other forms of insurance in their endorsement of any motivations. These univariate differences had minimal effect on the order of means across groups: across all demographics, reducing long-term risk remained the highest, symmetry remained the mid-rated reason, and avoiding treatment and avoiding future visits remained low-rated reasons.

**Table 2:** Mean comparisons of participant demographics among CPM motivations

	Patient Dichotomized Demographics											
	Diagnosis		Age		Education		Employment		Income		Insurance	
	Less	More	Low	High	Low	High	No	Yes	Low	High	Public	Private
CPM Motivations												
Avoiding Treatment	1.79 (1.15)	2.40 (1.30)**	1.76 (1.21)	2.38 (1.26)**	2.26 (1.26)	1.96 (1.21)	2.45 (1.32)	1.97 (1.18)^†^	2.46 (1.45)	2.08 (1.14)	1.81 (1.34)	2.16 (1.22)
Reducing Long-term Risk	4.38 (.91)	4.06 (.89)^†^	4.31 (.84)	4.11 (.95)	4.19 (.91)	4.18 (.93)	3.99 (.87)	4.29 (.93)	4.52 (.67)	4.03 (.98)**	4.15 (.94)	4.27 (.89)
Avoiding Future Visits	2.09 (1.36)	2.35 (1.14)	2.25 (1.30)	2.23 (1.21)	2.23 (1.10)	2.19 (1.35)	2.47 (1.09)	2.09 (1.27)	2.39 (1.09)	2.25 (1.27)	1.96 (1.15)	2.23 (1.26)
Symmetry	3.42 (1.53)	3.42 (1.39)	3.82 (1.42)	3.16 (1.41)*	3.05 (1.45)	3.77 (1.37)**	3.11 (1.37)	3.58 (1.45)	3.54 (1.70)	3.56 (1.27)	3.29 (1.71)	3.48 (1.41)

Means are presented and standard deviations are within parentheses. When relevant, significant mean differences are noted, ** *p* < .01, * *p* < .05, ^†^ *p* = .06

The third stage of analysis comprised a series of hierarchical multiple regression analyses testing for the independent effects of each predictor while controlling for the effects of the other five demographic predictors as well as participant race. This analysis was motivated by the moderate correlations between most of the predictor variables (see Table 3).

**Table 3:** Correlations between dichotomized predictors

	Age	Diagnosis	Education	Employment	Income
Age	1.00				
Diagnosis	−.16				
Education	−.30**	.13			
Employment	−.28**	−.02	.25**		
Income	−.03	.01	.28**	.23*	
Insurance	.00	−.06	.25**	.20*	.43**

Note. *p < .05, *p* < .01**

As shown in Table 4, avoiding treatment was predicted by higher age (*p* < .05), lower income (*p* < .05), and more severe diagnoses (*p* < .05). Reducing long-term risk was predicted by lower income (*p* < .01), and symmetry was predicted by higher education (*p* < .05). None of the predictors were significantly associated with avoiding future visits. Effects in the univariate analyses that did not achieve significance in the multiple regression analysis indicate effects for one variable that do not persist when the other predictor variables are controlled.

**Table 4:** Regression analysis of patient demographics and disease severity predicting CPM motivations

	CPM Motivations							
	Avoiding treatment		Reducing long-term risk		Avoiding future visits		Symmetry	
	β	*t*	β	*t*	β	*t*	β	*t*
Block 1								
Race	.07	.52	.13	1.29	−.05	−.49	.06	.58
Block 2								
Race	.13	1.24	.16	1.54	−.02	−.17	.06	.61
Age	.23	2.21*	−.08	−.78	−.04	−.39	−.13	−1.91
Education	.003	.03	−.13	−1.19	.04	.37	.26	2.32*
Employment	−.03	−.25	.17	1.58	−.15	−1.29	.14	1.23
Income	−.23	−2.00*	−.33	−2.84**	−.05	−.41	−.09	−.75
Insurance	.16	1.47	.15	1.35	.05	.42	−.06	−.51
Diagnosis	.24	2.36*	−.13	−1.31	.10	.92	.04	.40
Model								
Adj. R^2^	.12		.09		−.04		.06	
F	2.88**		2.33*		.49		1.91	

Note. *p < .05, *p* < .01**

## Discussion

This study shows statistically significant differences in CPM motivations as a function of age, education, income, and severity of diagnosis, but also shows considerable similarity in the motivations that are most important to women. Across socioeconomic differences, women participating in our study rated reducing future risk (including survival and avoiding a new primary in the non-involved breast) as the most powerful motivation for their decision to elect CPM. This pattern mirrors the results of several other studies showing that women’s foremost goal in choosing CPM is improving their long-term outcomes [8, 11]. The consistency of this motivation across demographic categories underscores a set of interrelated problems. Women perceive substantial risk from a new contralateral primary, even though that risk is objectively low [11, 28–30]. Further, they believe that CPM improves their long-term outcomes even though the medical evidence does not support this belief (except for women with BRCA mutations) [11]. Despite socioeconomic diversity, which is proxy for participation in different communities, exposure to different media, and different experiences with the health care system, breast cancer patients who choose CPM consistently express the same primary, and erroneous, motivation. Correspondingly, this study suggests that CPM patients are not responding rationally to evidence-based recommendations for more conservative treatment—or that their surgeons are not making those recommendations with sufficient strength.

In the context of this similarity, there were some notable differences in CPM motivations as a function of severity of diagnosis, income, age, and education.

### Severity of diagnosis

Compared to patients less severe disease, patients with invasive breast cancer were more motivated by avoiding treatment. Although both invasive breast cancers (more severe) and DCIS (less severe) can be treated by breast conservation or mastectomy, only patients with invasive disease will be offered chemotherapy and all of them will undergo some type of lymph node surgery. Patients with invasive disease face the prospect of more treatment over a longer period of time and their rationale for choosing CPM to avoid additional treatment seems to reflect this. Patients treated for DCIS have a relatively short course of treatment and thus their choice of CPM appears to depend more on wanting to avoid a new breast neoplasm than on avoiding treatment.

### Lower income

Controlling for education, employment, and insurance (along with age, diagnosis, and race), lower income predicted stronger motivations for CPM based on both long-term risk reduction and avoiding treatment. Thus, these income effects are *not* best explained as resulting from knowledge about breast cancer or CPM, or the direct costs of treatment, hospitalization, or surveillance. A survey of the literature reveals a paucity of data on the effects of a subsequent cancer diagnosis, i.e., recurrence or new primary breast cancer, on low income individuals and households. A possible explanation for the effect of income on CPM decision-making is that because lower-income women are essential to the financial stability of their households, they are especially concerned about the indirect costs of a new primary and associated treatments. Lower-income women in paid employment often have no sick leave; indeed, a 2015 report from the Bureau of Labor Statistics estimates that 39% of the private-sector workforce lacks paid sick days [31]. Unpaid work days deprive families of funds for rent, utilities, transportation, food and other critical expenses, and may result in job loss [32, 33]. Even when lower-income women do not work for pay, they often provide crucial domestic labor (including childcare and eldercare) that allows others to work outside the home [34, 35]. Thus, independent of their education, insurance, or employment, low-income women may have personal incentives to avoid time-consuming treatments for a contralateral new primary that will require substantial time away from normal activities for additional treatments. Since there were no questions in our survey specifically directed to concerns regarding lost jobs, lost income, or sick leave, this explanation for the influence of income will have to be directly tested in a future study.

### Age and education

Prior studies have noted that concerns about symmetry and the option for reconstruction prompt CPM [11, 23, 36, 37]. Indeed, King and colleagues found that patients were more likely to select CPM instead of other surgical options when they had strong symmetry concerns [37]. In the current study, not only was symmetry motivation the second-strongest, but patients who were younger and had more education more strongly endorsed symmetry as a motivation for CPM. The effect of age is consistent with greater general concern for physical appearance in younger women [38] and with results recently reported by Tesson and colleagues [39]. McLaren and Kuh’s analysis of data from the Medical Research Council National Survey of Health and Development suggests that appearance esteem is more sensitive to education than to adult occupationally-defined social class [40]. Correspondingly, more highly-educated women may be more concerned about the impact of breast cancer treatment on their physical appearance. Consequently, with both younger and more-educated women, physicians need to address alternatives to CPM with greater attention to appearance-related concerns.

### Limitations

This study exhibits several limitations that should be considered when interpreting our findings and conducting future research. Respondents self-reported their type of breast neoplasm; the acquisition of clinical data regarding the patients’ tumors, stage of disease, receipt of reconstruction, etc. was not a part of the approved protocol. Additionally, we did not inquire as to the respondents’ family history of breast cancer, number of children or desire for future children, or the disease status at the time of the interview. However, in a companion study to this one we did explore the influence of family and friends treated for breast cancer on the decision to undergo CPM [27]. These or other factors might explain greater variance in participants’ motivations than we were able to explain. We also have no data on the patients who we could not reach by telephone. The inability to reach these potential participants may have introduced bias into our sample. Further, our sample included fewer ethnic minority participants than desired; future prospective research efforts should consider collaboration with community leaders/groups to improve participant diversity [41, 42]. We did not specifically inquire as to BRCA 1/2 mutation carrier status, which raises the possibility that there were more patients (other than the four who identified themselves as carriers) that should have been excluded as CPM would have been indicated. Nonetheless, we believe this study adds important information relevant to the CPM debate. Most of the data regarding SES and its impact on the choice of CPM comes from large regional and national databases that tell us who undergoes CPM but not why. By querying women who chose CPM, we have obtained evidence that the “why?” does not differ greatly as a function of SES: patients are making many of the same assumptions about CPM, survival, and new primary, and thus making similar decisions.

## Conclusion

In summary, the current study highlights both consistency and variation in motivations for CPM—and points to the complexity of intervening to halt the trend in selecting this procedure. Across socioeconomic variation, women who choose CPM are highly motivated by the desire to reduce overall risk and risk of a new primary, and improve survival. This is not surprising, since many women overestimate the risk of developing contralateral breast cancer [14, 30]. Further, some women understand the relatively small risk of recurrence but nonetheless prize the small reduction in risk afforded by CPM [10, 11]. To help dissuade patients from CPM, physicians must provide careful explanation that the risk of distant metastases from the index lesion is much greater than the likelihood of a second primary breast cancer [43], and that metastatic disease not a new primary, will be responsible for patient death. There is a relatively robust portfolio of research on the perceived risk of breast cancer as a function of demographic and psychological variables, which has been summarized nicely in a meta-analysis by Katapodi and colleagues [21] and revisited more recently by Orom et al. [22]. Since statistical probabilities are often difficult for patients to understand, physicians should consider using visual aids that improve understanding of risk (e.g., depictions of frequency of recurrence per 100 or 1000 patients) [44]. Physicians also need to ensure that patients understand the potential adverse effects of CPM [45]. And, they need to help patients reduce the anxiety that contributes to exaggerated perceptions of risk and leads to surgical decisions that will not affect their survival [11, 43, 46]. Women also choose CPM to avoid to avoid breast asymmetry. A symmetrical appearance is an understandable goal, so physicians need to help patients realize that CPM is not necessary to achieve it. Finally, guiding treatment decisions for lower-income women may require that physicians compassionately address the tension between the minimal likelihood of a new primary and the real economic implications if it occurs.

## Additional file

Additional file 1: CPM Questionnaire. Questionnaire utilized to collect data for this study. (PDF 760 kb)
