# Supplementary material for: Motivations for contralateral prophylactic mastectomy as a function of socioeconomic status
Source: BMC Womens Health. 2017 Feb 1;17:10. doi: 10.1186/s12905-017-0366-2 (PMC5286852; doi:10.1186/s12905-017-0366-2)
Supplement: Additional file 1: — CPM Questionnaire. Questionnaire utilized to collect data for this study. (PDF 760 kb) [file 12905_2017_366_MOESM1_ESM.pdf]

Indiana Clinical and Translational Sciences Institute

CPM Questionnaire Study

Data Dictionary Codebook

04/10/2015 12:46pm

#	Variable / Field Name	Field Label<br>*Field Note*	Field Attributes (Field Type, Validation, Choices, Calculations, etc.)
Instrument: **Questionnaire**			
1	cpm	CPM#	text
2	actual_cpm	Actual CPM#	text
3	year_of_diagnosis	Year of diagnosis	text
4	age_at_initial_diagnosis	Age at initial diagnosis	text
5	relationship_status	Relationship status	radio<br>1 Married<br>2 Single<br>3 Widowed<br>4 Divorced/separated<br>5 Domestic partnership<br>6 declined to answer
6	race	Race	radio<br>1 Black/African descent<br>2 Caucasian<br>3 Hispanic<br>4 Asian<br>5 Pacific Islander<br>6 American Indian/Alaska Native<br>7 Other<br>8 declined to answer
7	recall_diagnosis	Recall Diagnosis	radio

			1 DCIS<br>2 Invasive ductal cancer<br>3 Invasive lobular cancer<br>4 Invasive cancer<br>5 Mass/lump<br>6 Mammogram abnormality<br>7 Other<br>8 Does not recall<br>9 declined to answer<br>10 nipple discharge
8	bcs_an_option	BCS option offered	yesno<br>1 Yes<br>0 No
9	bcs_not_an_option_because	Why was BCS not an option	checkbox<br>1 bcs_not_an_option_because___1 Tumor too large<br>2 bcs_not_an_option_because___2 Breasts too small<br>3 bcs_not_an_option_because___3 BRCA gene mutation known prior to surgery<br>4 bcs_not_an_option_because___4 Surgeon opposed to BCS without concrete reason<br>5 bcs_not_an_option_because___5 Extensive DCIS<br>8 bcs_not_an_option_because___8 Multiple masses/lumps present<br>6 bcs_not_an_option_because___6 Other<br>7 bcs_not_an_option_because___7 Not applicable

			9 | bcs_not_an_option_because___9 | does not recall why<br>10 | bcs_not_an_option_because___10 | skin involvement
10	reasons_for_cm_over_bcs	Reasons for choosing CM over BCS	notes
11	reason_for_cm_over_bcs	Chemotherapy	radio (Matrix)<br>1 | Not at all<br>2 | A very small amount<br>3 | A moderate amount<br>4 | A large amount<br>5 | A very large amount<br>6 | Not applicable as BCS not an option/BCS not offered<br>7 | declined to answer
12	reason_for_cm_over_bcs2	Radiation	radio (Matrix)<br>1 | Not at all<br>2 | A very small amount<br>3 | A moderate amount<br>4 | A large amount<br>5 | A very large amount<br>6 | Not applicable as BCS not an option/BCS not offered<br>7 | declined to answer
13	reason_for_cm_over_bcs3	Medications	radio (Matrix)<br>1 | Not at all<br>2 | A very small amount<br>3 | A moderate amount<br>4 | A large amount<br>5 | A very large amount<br>6 | Not applicable as BCS not an option/BCS not offered<br>7 | declined to answer

14	reason_for_cm_over_bcs4	Future procedures	radio (Matrix)<br>1 Not at all<br>2 A very small amount<br>3 A moderate amount<br>4 A large amount<br>5 A very large amount<br>6 Not applicable as BCS not an option/BCS not offered<br>7 declined to answer
15	reason_for_cm_over_bcs5	Recurrence in ipsilateral breast	radio (Matrix)<br>1 Not at all<br>2 A very small amount<br>3 A moderate amount<br>4 A large amount<br>5 A very large amount<br>6 Not applicable as BCS not an option/BCS not offered<br>7 declined to answer
16	reason_for_cm_over_bcs6	Recurrence in contralateral breast	radio (Matrix)<br>1 Not at all<br>2 A very small amount<br>3 A moderate amount<br>4 A large amount<br>5 A very large amount<br>6 Not applicable as BCS not an option/BCS not offered<br>7 declined to answer
17	reason_for_cm_over_bcs7	Metastasis	radio (Matrix)<br>1 Not at all<br>2 A very small amount<br>3 A moderate amount

			4 A large amount<br>5 A very large amount<br>6 Not applicable as BCS not an option/BCS not offered<br>7 declined to answer
18	reason_for_cm_over_bcs8	CM better than BCS for risk reduction	radio (Matrix)<br>1 Not at all<br>2 A very small amount<br>3 A moderate amount<br>4 A large amount<br>5 A very large amount<br>6 Not applicable as BCS not an option/BCS not offered<br>7 declined to answer
19	reason_for_cm_over_bcs9	CM better than BCS at prolonging survival	radio (Matrix)<br>1 Not at all<br>2 A very small amount<br>3 A moderate amount<br>4 A large amount<br>5 A very large amount<br>6 Not applicable as BCS not an option/BCS not offered<br>7 declined to answer
20	reason_for_cm_over_bcs10	Reduce or avoid physician visits	radio (Matrix)<br>1 Not at all<br>2 A very small amount<br>3 A moderate amount<br>4 A large amount<br>5 A very large amount<br>6 Not applicable as BCS not an option/BCS not offered<br>7 declined to answer

21	reason_for_cm_over_bcs11	Reduce or avoid future surgery	radio (Matrix)<br>1 Not at all<br>2 A very small amount<br>3 A moderate amount<br>4 A large amount<br>5 A very large amount<br>6 Not applicable as BCS not an option/BCS not offered<br>7 declined to answer
22	perceived_bcs_recurrence	Perceived risk of recurrence with BCS	text
23	perceived_contralateral_risk	Perceived contralateral risk	text
24	perceived_metastasis_risk	Perceived metastasis risk	text
25	risk_perception1	CM reduces likelihood of recurrence	radio (Matrix)<br>1 Strongly disagree<br>2 Disagree<br>3 Uncertain<br>4 Agree<br>5 Strongly agree<br>6 Not applicable as BCS not an option<br>7 declined to answer
26	risk_perception2	BCS reduces likelihood of recurrence	radio (Matrix)<br>1 Strongly disagree<br>2 Disagree<br>3 Uncertain<br>4 Agree<br>5 Strongly agree<br>6 Not applicable as BCS not an option<br>7 declined to answer
27	delayed_versus_immediate_cpm	Immediate versus delayed CPM	radio<br>1 At the time of the original surgery

			2 At a second later surgery<br>3 Declined to answer
28	cpm_reasons	Reasons for CPM	notes
29	general_cpm_reasons1	Chemotherapy	radio (Matrix)<br>1 Not at all<br>2 A very small amount<br>3 A moderate amount<br>4 A large amount<br>5 A very large amount<br>6 Not applicable/declined to answer
30	general_cpm_reasons2	Radiation	radio (Matrix)<br>1 Not at all<br>2 A very small amount<br>3 A moderate amount<br>4 A large amount<br>5 A very large amount<br>6 Not applicable/declined to answer
31	general_cpm_reasons3	Medications (eg. Tamoxifen, Arimidex)	radio (Matrix)<br>1 Not at all<br>2 A very small amount<br>3 A moderate amount<br>4 A large amount<br>5 A very large amount<br>6 Not applicable/declined to answer
32	general_cpm_reasons4	Future procedures like MRI, or mammograms	radio (Matrix)<br>1 Not at all<br>2 A very small amount<br>3 A moderate amount<br>4 A large amount<br>5 A very large amount

			6 Not applicable/declined to answer
33	general_cpm_reasons5	Concern for contralateral breast cancer	radio (Matrix)<br>1 Not at all<br>2 A very small amount<br>3 A moderate amount<br>4 A large amount<br>5 A very large amount<br>6 Not applicable/declined to answer
34	general_cpm_reasons6	Concern for metastasis	radio (Matrix)<br>1 Not at all<br>2 A very small amount<br>3 A moderate amount<br>4 A large amount<br>5 A very large amount<br>6 Not applicable/declined to answer
35	general_cpm_reasons7	Believe that CPM reduces recurrence	radio (Matrix)<br>1 Not at all<br>2 A very small amount<br>3 A moderate amount<br>4 A large amount<br>5 A very large amount<br>6 Not applicable/declined to answer
36	general_cpm_reasons8	Belief that CPM increases long term survival	radio (Matrix)<br>1 Not at all<br>2 A very small amount<br>3 A moderate amount<br>4 A large amount<br>5 A very large amount<br>6 Not applicable/declined to answer
37	general_cpm_reasons9	To maintain a symmetrical appearance	radio (Matrix)

			1 Not at all<br>2 A very small amount<br>3 A moderate amount<br>4 A large amount<br>5 A very large amount<br>6 Not applicable/declined to answer
38	general_cpm_reasons10	To avoid future physician visits	radio (Matrix)<br>1 Not at all<br>2 A very small amount<br>3 A moderate amount<br>4 A large amount<br>5 A very large amount<br>6 Not applicable/declined to answer
39	general_cpm_reasons11	To reduce or void later breast surgeries	radio (Matrix)<br>1 Not at all<br>2 A very small amount<br>3 A moderate amount<br>4 A large amount<br>5 A very large amount<br>6 Not applicable/declined to answer
40	friend_family1	Grandmother	radio (Matrix)<br>5 0- did not influence at all<br>1 1<br>2 2<br>3 3<br>4 4<br>6 5- significantly influenced<br>7 Not applicable<br>8 declined to answer
41	friend_family2	Mother	radio (Matrix)

				5 0- did not influence at all<br>1 1<br>2 2<br>3 3<br>4 4<br>6 5- significantly influenced<br>7 Not applicable<br>8 declined to answer
42	friend_family3	Sister		radio (Matrix)<br>5 0- did not influence at all<br>1 1<br>2 2<br>3 3<br>4 4<br>6 5- significantly influenced<br>7 Not applicable<br>8 declined to answer
43	friend_family4	Aunt		radio (Matrix)<br>5 0- did not influence at all<br>1 1<br>2 2<br>3 3<br>4 4<br>6 5- significantly influenced<br>7 Not applicable<br>8 declined to answer
44	friend_family5	Cousin		radio (Matrix)<br>5 0- did not influence at all<br>1 1<br>2 2

			3 3<br>4 4<br>6 5- significantly influenced<br>7 Not applicable<br>8 declined to answer
45	friend_family6	Friend1	radio (Matrix)<br>5 0- did not influence at all<br>1 1<br>2 2<br>3 3<br>4 4<br>6 5- significantly influenced<br>7 Not applicable<br>8 declined to answer
46	friend_family7	Friend2	radio (Matrix)<br>5 0- did not influence at all<br>1 1<br>2 2<br>3 3<br>4 4<br>6 5- significantly influenced<br>7 Not applicable<br>8 declined to answer
47	friend_family8	Friend3	radio (Matrix)<br>5 0- did not influence at all<br>1 1<br>2 2<br>3 3<br>4 4<br>6 5- significantly influenced

			7 Not applicable<br>8 declined to answer
48	friend_family12	Partner	radio (Matrix)<br>5 0- did not influence at all<br>1 1<br>2 2<br>3 3<br>4 4<br>6 5- significantly influenced<br>7 Not applicable<br>8 declined to answer
49	friend_family13	Father	radio (Matrix)<br>5 0- did not influence at all<br>1 1<br>2 2<br>3 3<br>4 4<br>6 5- significantly influenced<br>7 Not applicable<br>8 declined to answer
50	friend_family14	Uncle	radio (Matrix)<br>5 0- did not influence at all<br>1 1<br>2 2<br>3 3<br>4 4<br>6 5- significantly influenced<br>7 Not applicable<br>8 declined to answer

51	friend_family15	Brother	radio (Matrix)<br>5 0- did not influence at all<br>1 1<br>2 2<br>3 3<br>4 4<br>6 5- significantly influenced<br>7 Not applicable<br>8 declined to answer
52	friend_family16	Friend 4	radio (Matrix)<br>5 0- did not influence at all<br>1 1<br>2 2<br>3 3<br>4 4<br>6 5- significantly influenced<br>7 Not applicable<br>8 declined to answer
53	friend_family17	Friend 5	radio (Matrix)<br>5 0- did not influence at all<br>1 1<br>2 2<br>3 3<br>4 4<br>6 5- significantly influenced<br>7 Not applicable<br>8 declined to answer
54	friend_family18	Friend 6	radio (Matrix)<br>5 0- did not influence at all<br>1 1

			2 2<br>3 3<br>4 4<br>6 5- significantly influenced<br>7 Not applicable<br>8 declined to answer
55	friend_family_influence	How friends/family influenced choice	notes
56	other_influence9	Children	radio (Matrix)<br>5 0-did not influence at all<br>1 1<br>2 2<br>3 3<br>4 4<br>6 5- significantly influenced<br>7 Not applicable<br>8 declined to answer
57	other_influence10	Parent(s)	radio (Matrix)<br>5 0-did not influence at all<br>1 1<br>2 2<br>3 3<br>4 4<br>6 5- significantly influenced<br>7 Not applicable<br>8 declined to answer
58	other_influence11	Sibling(s)	radio (Matrix)<br>5 0-did not influence at all<br>1 1<br>2 2

			3 3<br>4 4<br>6 5- significantly influenced<br>7 Not applicable<br>8 declined to answer
59	other_influence12	Cousin(s)	radio (Matrix)<br>5 0-did not influence at all<br>1 1<br>2 2<br>3 3<br>4 4<br>6 5- significantly influenced<br>7 Not applicable<br>8 declined to answer
60	other_influence13	Aunt(s)	radio (Matrix)<br>5 0-did not influence at all<br>1 1<br>2 2<br>3 3<br>4 4<br>6 5- significantly influenced<br>7 Not applicable<br>8 declined to answer
61	other_influence14	Uncle(s)	radio (Matrix)<br>5 0-did not influence at all<br>1 1<br>2 2<br>3 3<br>4 4<br>6 5- significantly influenced

			7 Not applicable<br>8 declined to answer
62	other_influence15	Grandparent(s)	radio (Matrix)<br>5 0-did not influence at all<br>1 1<br>2 2<br>3 3<br>4 4<br>6 5- significantly influenced<br>7 Not applicable<br>8 declined to answer
63	other_influence16	Surgeon	radio (Matrix)<br>5 0-did not influence at all<br>1 1<br>2 2<br>3 3<br>4 4<br>6 5- significantly influenced<br>7 Not applicable<br>8 declined to answer
64	other_influence17	Oncologist	radio (Matrix)<br>5 0-did not influence at all<br>1 1<br>2 2<br>3 3<br>4 4<br>6 5- significantly influenced<br>7 Not applicable<br>8 declined to answer
65	other_influence18	Psychiatrist	radio (Matrix)

			5 0-did not influence at all<br>1 1<br>2 2<br>3 3<br>4 4<br>6 5- significantly influenced<br>7 Not applicable<br>8 declined to answer
66	other_influence19	Ob/Gyn	radio (Matrix)<br>5 0-did not influence at all<br>1 1<br>2 2<br>3 3<br>4 4<br>6 5- significantly influenced<br>7 Not applicable<br>8 declined to answer
67	other_influence20	Primary care physician	radio (Matrix)<br>5 0-did not influence at all<br>1 1<br>2 2<br>3 3<br>4 4<br>6 5- significantly influenced<br>7 Not applicable<br>8 declined to answer
68	other_influence21	Nurse(s)	radio (Matrix)<br>5 0-did not influence at all<br>1 1

			2 2<br>3 3<br>4 4<br>6 5- significantly influenced<br>7 Not applicable<br>8 declined to answer	
69	other_influence22	Other health care professional	radio (Matrix)<br>5 0-did not influence at all<br>1 1<br>2 2<br>3 3<br>4 4<br>6 5- significantly influenced<br>7 Not applicable<br>8 declined to answer	
70	other_influence23	Other cancer survivor	radio (Matrix)<br>5 0-did not influence at all<br>1 1<br>2 2<br>3 3<br>4 4<br>6 5- significantly influenced<br>7 Not applicable<br>8 declined to answer	
71	other_influence24	Pastor/Priest	radio (Matrix)<br>5 0-did not influence at all<br>1 1<br>2 2<br>3 3<br>4 4	

			6 5- significantly influenced<br>7 Not applicable<br>8 declined to answer
72	other_influence25	Partner/Spouse	radio (Matrix)<br>5 0-did not influence at all<br>1 1<br>2 2<br>3 3<br>4 4<br>6 5- significantly influenced<br>7 Not applicable<br>8 declined to answer
73	other_influence26	Friend	radio (Matrix)<br>5 0-did not influence at all<br>1 1<br>2 2<br>3 3<br>4 4<br>6 5- significantly influenced<br>7 Not applicable<br>8 declined to answer
74	influence_of_others	Influence of others including spouse, health care providers or clergy	notes
75	support_group_participation	Do you participate in support groups	yesno<br>1 Yes<br>0 No
76	support_group_influence1	Church group	radio (Matrix)<br>5 0- did not influence at all<br>1 1<br>2 2

			3 3<br>4 4<br>6 5-significantly influenced<br>7 Not applicable<br>8 declined to answer
77	support_group_influence2	Sorority sisters	radio (Matrix)<br>5 0- did not influence at all<br>1 1<br>2 2<br>3 3<br>4 4<br>6 5-significantly influenced<br>7 Not applicable<br>8 declined to answer
78	support_group_influence3	Breast cancer survivor group	radio (Matrix)<br>5 0- did not influence at all<br>1 1<br>2 2<br>3 3<br>4 4<br>6 5-significantly influenced<br>7 Not applicable<br>8 declined to answer
79	support_group_influence4	Social outing group	radio (Matrix)<br>5 0- did not influence at all<br>1 1<br>2 2<br>3 3<br>4 4

			6 5-significantly influenced<br>7 Not applicable<br>8 declined to answer
80	support_group_influence5	Other	radio (Matrix)<br>5 0- did not influence at all<br>1 1<br>2 2<br>3 3<br>4 4<br>6 5-significantly influenced<br>7 Not applicable<br>8 declined to answer
81	support_group_influence	How did support groups influence choice	notes
82	media_influence	Do you recall any media specific information around the time of your diagnosis	yesno<br>1 Yes<br>0 No
83	specific_media_item_of_influe nce	What media specific information do you recall around the time of your diagnosis	notes
84	media_influence1	TV	radio (Matrix)<br>5 0-did not influence at all<br>1 1<br>2 2<br>3 3<br>4 4<br>6 5-significantly influenced<br>7 Not applicable<br>8 declined to answer
85	media_influence2	Radio	radio (Matrix)<br>5 0-did not influence at all

			1 1<br>2 2<br>3 3<br>4 4<br>6 5-significantly influenced<br>7 Not applicable<br>8 declined to answer
86	media_influence3	Internet	radio (Matrix)<br>5 0-did not influence at all<br>1 1<br>2 2<br>3 3<br>4 4<br>6 5-significantly influenced<br>7 Not applicable<br>8 declined to answer
87	media_influence4	Books/Magazines	radio (Matrix)<br>5 0-did not influence at all<br>1 1<br>2 2<br>3 3<br>4 4<br>6 5-significantly influenced<br>7 Not applicable<br>8 declined to answer
88	surgical_discussion	Description of surgical discussion	notes
89	surgeon_support	Did your surgeon suggest the surgery you selected	yesno<br>1 Yes<br>0 No

90	final_surgeon_support	Was your surgeon supportive of your final choice	yesno<br>1 Yes<br>0 No
91	patient_influence_on_surgeon	How did you change your surgeon's mind	notes
92	surgeon_trust	How much did you trust your surgeon's information	radio<br>1 Not at all<br>2 A little bit<br>3 Somewhat<br>4 Quite a bit<br>5 Completely<br>6 declined to answer
93	lack_of_surgeon_trust	What did you not trust about your surgeon's information (Either actual or alt opinion surgeon)	notes
94	second_surgical_opinion	Did you seek an opinion from any other surgeon	yesno<br>1 Yes<br>0 No
95	education_level	Highest level of education	radio<br>1 Some Highschool or less<br>2 Highschool diploma/GED<br>3 Some college/Associate's degree<br>4 Bachelor's degree<br>5 Master's degree<br>6 Ph.D./JD/MD<br>7 Other<br>8 declined to answer
96	employment_status	Employment status	radio<br>1 Paid worker<br>2 Homemaker<br>3 Student<br>4 Retired<br>5 Disabled

			6 Unemployed<br>7 Other<br>8 declined to answer
97	occupation	Occupation	text
98	fulltime_vs_partime	Full or part time employment	radio<br>1 Full time<br>2 Part time<br>3 Not applicable
99	no_of_people_in_home	How many people reside in your household	text
100	head_of_household	Who was the head of your household	radio<br>1 Self<br>2 Spouse<br>3 Parent<br>4 Other<br>5 declined to answer
101	other_description	If other please describe	text
102	gross_income	Gross income	radio<br>1 0 to 25000<br>2 25000 to 50000<br>3 50000 to 75000<br>4 75000 to 100000<br>5 100000 or more<br>6 unknown<br>7 declined to answer
103	medical_insurance	Insurance type	radio<br>1 Private<br>2 Medicare<br>3 Medicaid<br>4 No insurance<br>5 Other

			6 declined to answer
104	insurance_other_description	If other please describe	text
105	questionnaire_complete	Complete?	dropdown<br>0 Incomplete<br>1 Unverified<br>2 Complete
